# Determination of mitomycin C in rat plasma by liquid chromatography-tandem mass spectrometry and its application for determining pharmacokinetics in rat

**DOI:** 10.1016/j.heliyon.2024.e32927

**Published:** 2024-06-13

**Authors:** Kyung-Sun Moon, Jong-Min Song, JiMin Yi, Quynh Khoa Pham, Sung-Hoon Ahn

**Affiliations:** Laboratory of Pharmaceutical Sciences, College of Pharmacy, Kangwon National University, Chuncheon, Gangwondo, 24341, Republic of Korea

**Keywords:** LC-MS/MS, Mitomycin C, Pharmacokinetics

## Abstract

To develop the liquid chromatography-tandem mass spectrometry (LC-MS/MS) method for measuring mitomycin C in rat plasma, samples were processed using solid-phase extraction, with the internal standard being carbamazepine. A reversed phased C18 column was utilized for the LC-MS/MS study, and mobile phases consisting of 0.1 % formic acid in acetonitrile and water were injected into it at a rate of 0.3 mL/min. Multiple reaction monitoring in positive-ion mode with precursor-product ion pairs 335.3 → 242.3 (mitomycin C) and 237.1 → 194.1 (carbamazepine) was employed to quantify the compounds. The linear range in plasma was found to be 10–4000 ng/mL (r^2^ = 0.992). The inter-batch and intra-batch precision were <14.3 % (LLOQ: 14.7 %) and 13.4 % (LLOQ: 16.1 %), respectively. The recovery and the matrix effect of mitomycin C in plasma were 113 % and 111 %, respectively. Mitomycin C was stable under the conditions of this assay method. In the end, this approach proved effective in a pharmacokinetic investigation with the intravenous and oral administration of mitomycin C to rats.

## Highlights

• We develop an assay method for mitomycin C in rat plasma using LC-MS/MS.
• We develop small quantity needed, rapid, and sensitive method by solid phase extraction.
• This assay variability limits set forth in the FDA guidelines.
• The present method can be applied to pharmacokinetic and/or toxicokinetic studies.

## 1 Introduction

Mitomycin C (MMC) is a chemotherapeutic agent, found in *Streptomyces caespitosus* [[1], [2], [3], [4]]. MMC is a potent alkylating agent and DNA crosslinker, which in turn prevents protein synthesis by inhibiting DNA transcription into RNA [5]. To be more specific, MMC is first reduced to form a mitosene, and this product is then linked to DNA's guanine nucleotide through a series of alkylation steps, leading to forming covalent cross-links with other guanine nucleotides [6,7]. Consequently, the DNA double helix is prevented from unwinding and separating, inhibiting cell proliferation [6]. To date, MMC is widely used in the treatment of bladder, colorectal, and stomach cancers and is effective in reducing tumor recurrence rates in low- and intermediate-risk tumors [[8], [9], [10], [11]].

MMC contains three functional groups, namely phenylhydrazine, urethane, and ethyleneimine [8,12]. Main side effect of MMC is bone marrow suppression, which can result in serious blood issues such as leukopenia and low WBC counts (low platelet levels). In addition, mitomycin C raises the possibility of suffering from hemolytic uremic syndrome [13,14]. But in various situations, especially when there are not equally effective or less harmful substitutes, taking mitomycin C may be seen to outweigh the negative effects. MMC is being used as an adjuvant in the management of glaucoma, colorectal cancer, and non-muscle invasive bladder cancer [[15], [16], [17]]. Although MMC can be administered orally [18], it has been mainly used for intraperitoneal or intravesical administration in various studies due to its rapid metabolism in liver. In previous studies, it has reported a half-life of 1.35 ± 0.15 h and a distribution volume at a steady state of 0.13 ± 0.01 L/kg for MMC [19]. According to tissue distribution study, MMC was distributed in heart, liver, kidney, lung, and spleen [20]. The unmetabolized form of MMC is rapidly excreted through bile and urine, with 1.8 % of biliary excretion being reabsorbed [21,22].

Until now, there have been several reports using different analytical assays such as high-performance liquid chromatography (HPLC) with ultraviolet–visible spectroscopy (UV–Vis) or a diode array detector (DAD) for the quantification of MMC in biological samples [[23], [24], [25], [26], [27], [28], [29]]. Although liquid chromatography-tandem mass spectrometry (LC-MS/MS) was also used to determine MMC in biological fluids and even on various vinyl and stainless steel surfaces [[30], [31], [32]], these analytical methods were not sufficiently rapid and sensitive for application of further pharmacokinetic studies using animal models. Among these assays, HPLC-MS/MS-based analytical methods recently showed more robust, selectivity and sensitivity than the others. In addition, multiple reaction monitoring (MRM) used in LC-MS/MS assay can provide high selectivity and specificity specific ions selected by a particular precursor ion and collision-induced fragmental ion, so MRM is a very powerful method for measuring drug exposure in biological fluids. The aim of this investigation was to create and verify an accurate and focused LC-MS/MS methodology for determining out MMC concentrations in rat plasma. The developed and validated assay method of MMC for pharmacokinetic studies has the potential to be further applied in toxicokinetic, pharmacological, and nonclinical studies to ensure the safe use of MMC.

## 2 Experimental

### 2.1 Chemicals and reagents

Mitomycin C (MMC, (4*S*,6*S*,7*R*,8*S*)-11-amino-7-methoxy-12-methyl-10, >98 %), carbamazepine (CBZ, >98 %), polyethylene glycol (PEG) 400, dimethyl sulfoxide (DMSO), and formic acid were purchased from Sigma-Aldrich (St. Louis, MO, USA). Methanol (MeOH, >99.9 %) and Acetonitrile (ACN, >99.5 %) were from Fisher Scientific (Hampton, NH, USA). The Milli-Q system (Millipore, Bedford, MA, USA) provided the deionization water.

### 2.2 Stock solution, calibration standards, and quality control (QC) samples

Stock solutions of MMC and CBZ (internal standard, IS) were formed by dissolving each chemical individually at 1 mg/mL in DMSO. CBZ was selected as the IS due to its chemical stability during chromatographic separation and mass spectrometry detection, lack of interference with MMC analysis, absence in blank samples, and cost-effectiveness. Calibration standards and QC samples were prepared from a stock solution. Working standard solutions were prepared by serial dilution from stock solution with ACN. The IS solution was 200 ng/mL in 1:9 MeOH–H_2_O. All stock solutions were stored at −80 °C until use. And working solutions were stored in a refrigerator (−20 °C). Seven calibrators were prepared at nominal plasma concentrations of 10, 30, 100, 500, 1000, 2000, and 4000 ng/mL for MMC. Four levels of plasma QC samples (i.e. LLOQ, Low-, Mid-, High QCs) were prepared at 10, 30, 100, and 4000 ng/mL for analytes. Calibration standard and QC sample were regularly prepared by spiking 5 μL of the working solution with 45 μL of the blank rat plasma before analysis.

### 2.3 Instrumentation and chromatographic conditions

**Fig. 1:**
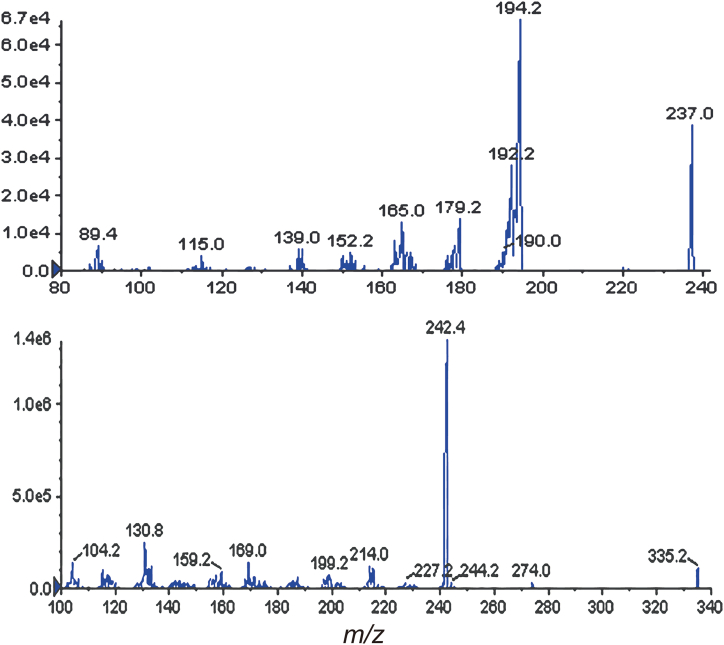
The mass spectra of MMC (A) and CBZ (B).

For the quantitative measurement of MMC in order to obtain positive ions, an electrospray ionization (ESI) interface coupled with a mass spectrometer API3200 (AB Sciex, Foster City, CA, USA) was utilized. The mass spectrometry conditions of MMC and IS were optimized in the positive ion mode. The primary fragmentation of the chemicals on the production mass spectra shown in Fig. 1 were used to quantify MMC. The C18 column possesses capabilities in separating hydrophobic compounds, and given the hydrophobic nature of mitomycin C's cyclic structure, analysis using C18 proves to be straightforward and efficient. A Gemini-NX-C18 column (100 mm × 2 mm, 3 μm; Phenomenex, Torrance, CA, USA) protected by a Security Guard C18 guard column (4 mm × 20 mm, Phenomenex, USA) was used for the chromatographic separation of the analytes. The autosampler was set to 4 °C, while the column temperature was 40 °C. The temperature of column oven was selected due to the favorable efficiency of mitomycin C resolution at 40 °C, ensuring maintenance of peak reproducibility without compromising on analysis time. Mobile phase A was 0.1 % of formic acid in water, and mobile phase B was 0.1 % of formic acid in ACN. The mobile phase running program was initiated at 10 % mobile phase B and maintained for the first 0.2 min, then linearly increased to 90 % mobile phase B at 1.5 min, followed by holding up to 90 % for 2 min; from 3.5 to 3.6 min, mobile phase gradient was back down to 10 % mobile phase B and from 3.6 min to 5 min returned to the initial status. There was a constant flow rate of 0.3 mL/min. Using target ions at *m*/*z* 237.1 → 194.1 for IS and *m*/*z* 335.3 → 242.3 for MMC, the mass spectrometer was operating in MRM mode of operation. The optimized instrument conditions were as follows: ion spray voltage, 5500 V; source temperature, 550 °C; curtain gas, 10 psi; nebulizing gas, 50 psi; heating gas, 50 psi; collision energy, 35 V for MMC and 29 V for IS. The analytical data were processed with Analyst software 1.4.2 (AB Sciex).

### 2.4 Solid phase extraction for plasma sample preparation

An Oasis HLB 1 cc Vac Cartridge was first rinsed with 1 mL of MeOH, followed by 1 mL of water immediately before used for extraction of the analytes and IS from rat plasma. Considering the amphiphilic property of MMC, the sample preparation step using HLB cartridges for SPE was selected because it would effectively separate the amphiphilic compounds from the biological fluids. An aliquot of 50 μL plasma sample (calibrators, QC samples, or plasma samples obtained from pharmacokinetic experiments), after being mixed with 50 μL of the IS working solution, then loaded onto the pre-conditioned SPE cartridge. First, the sample passed through the cartridge with minimum positive pressure, then was washed with 1 mL water. Finally, the analytes and IS were eluted with 1 mL MeOH into a clean microtube. For 2 h, the solvent evaporated under a nitrogen gas stream. The dehydrated residue was reconstituted by vortexing and mixing for 1 min with 100 μL 80 % acetonitrile in water containing 0.1 % formic acid. The resultant material was injected into a 5 μL aliquot for LC-MS/MS analysis.

### 2.5 Method validation

In compliance with FDA bioanalytical method validation guidelines, the developed method was validated [33]. Parameters validated included calibration curve and linearity, specificity and selectivity, carry-over, accuracy, precision, extraction recovery, matrix effect, and stability. Sample size was n = 4∼5 to validate the analytical method for the plasma concentration of MMC and comparative statistical method was used.

#### 2.5.1 Calibration curve and linearity

The standard calibration curve batches were prepared every analysis for linear evaluation. Calibration curve samples were prepared freshly on the day of analysis. Each calibration curve included double blank samples (without analytes or IS) and a single blank sample (only IS). Seven concentration levels (10, 30, 100, 500, 1000, 2000 and 4000 ng/mL) were used; the linearity range of plasma was between 10 and 4000 ng/mL. The calibration curve was generated by the response of peak area ratio (y) and analytes concentration (x), and the linear 1/X2 weighted relation was used for regression. With the exception of LLOQ, where 80–120 % was considered appropriate, the linearity of the calibration curves was judged acceptable when the back-calculated concentrations of each standard point fell within 85–115 % of their nominal concentrations.

#### 2.5.2 Selectivity and specificity

The selectivity of the assay method for endogenous plasma matrix components was assessed by extracting six separate blank plasma samples with or without additional IS and analytes. For the selectivity test to be acceptable, none of the six individual blank plasma could show an interference peak area at the retention time of the analytes that was >20.0 % of the mean analytes peak area from the LLOQ and none of the six individual blank plasma could show an interference peak area at the retention time of IS that was >5.0 % of the mean IS peak area. Chromatograms from blank plasma spiked at LLOQ levels were used to examine the specificity of the method and confirm that there were no interfering compounds around the retention time of analytes.

#### 2.5.3 Carry-over

Carry-over of analytes and IS was evaluated by analyzing an extracted blank sample directly after the ULOQ samples. The analytes peak area in the blank samples had to be no greater than 20 % of the lowest concentration sample's peak area on the standard curve. The IS peak area of the blank sample was required to be no more than 5 % of the IS peak area of the ULOQ samples.

#### 2.5.4 Accuracy and precision

By evaluating five QC replicates at four distinct concentrations (LLOQ, QCL, QCM, and QCH; 0.01, 0.03, 1, and 4 μg/mL) on one day and for five other days, respectively, the method's accuracy and precision were assessed. To assess accuracy, the mean concentrations of the analytes were compared to their nominal concentrations; a difference of no more than ±15 % from the nominal values was considered acceptable. With the exception of the LLOQ, where 20 % was acceptable for both accuracy and precision, the coefficient of variation (CV) values were computed to determine the precision; values could not exceed 15 %.

#### 2.5.5 Extraction recovery and matrix effect

Three sets of standards at low and high concentrations were analyzed in order to evaluate the extraction recovery and matrix influence. Set1 is the peak area of the analytes with reference concentrations in 10 % MeOH, Set2 is the peak area of the analytes with reference concentrations for the sample spiked with the target compounds after extraction, and Set3 is the peak area of the analytes with reference concentrations for the sample spiked with the target compounds before extraction [34,35].

• Matrix effect = $\left(\frac{set2}{set1}\right)$*100=(Post spiking analyte peak area/Neat analyte peak area)*100
• Recovery = $\left(\frac{set3}{set2}\right)$*100=(Pre spiking analyte peak area/Post analyte peak area)*100

All samples were analyzed in quintuples at each concentration level.

#### 2.5.6 Stability

This stability testing is important to ensure that the measured concentrations of MMC in rat plasma are not affected by different storage and handling conditions. The bench-top stability test assesses the impact of short-term storage at room temperature, whereas the freeze-thaw stability test assesses the impact of repeated cycles of freezing and thawing on the stability of MMC in rat plasma. The long-term stability test at −20 °C evaluates the effect of prolonged storage.

The acceptance criteria for stability testing are set at ±15 % deviation from the nominal concentrations for both low- and high-concentration QC samples, an CV of ≤15 % for each concentration level. These criteria ensure that the measured concentrations are within an acceptable range and that the precision of the method is maintained within the fixed times.

### 2.6 Pharmacokinetic study in rats

The pharmacokinetic properties of MMC in 9 week old male Sprague-Dawley (SD) rats whose weight was around 300 g were investigated using the validated method to measure its plasma concentrations. Prior to the trial, the rats were housed in the College of Pharmacy's animal room at Kangwon National University for at least of one week. They were also starved for the entire night before receiving MMC orally.

For both oral and intravenous administration, the dosage solutions were produced at a concentration of 5 mg/mL in PEG 400-distilled water-DMSO (40:55:5, v/v/v). Rat blood samples were taken via the femoral vein at 2, 10, 30 min, 1, 2, 4, 8, and 24 h followed the intravenous administration of 5 mg/kg of MMC and at 15, 30 min, 1, 2, 4, 8, and 24 h following the oral administration of 5 mg/kg of MMC. After immediately centrifuging the blood samples for 10 min at 4 °C (13000 rpm), the supernatants were moved to fresh containers and kept at −80 °C until analysis. Pharmacokinetic parameters such as C_max_, T_max_, K_e_, AUC_0→t_, AUC_0→∞_, and t_1/2_ were calculated. While K_e_ was calculated using the slope of the linear connection between the loge concentration and time during the terminal elimination phase, C_max_ and T_max_ values were directly obtained from the raw data. The linear trapezoidal formula was used to determine AUC_0→t_, and the ratio of the last measured plasma concentration to Ke was used to obtain AUC_0→∞_ after extrapolating from time zero to infinity. Based on the elimination rate constant, the elimination half-life was calculated as ln2/K_e_.

## 3 Results and discussion

### 3.1 Method development for LC-MS/MS analysis

**Fig. 2:**
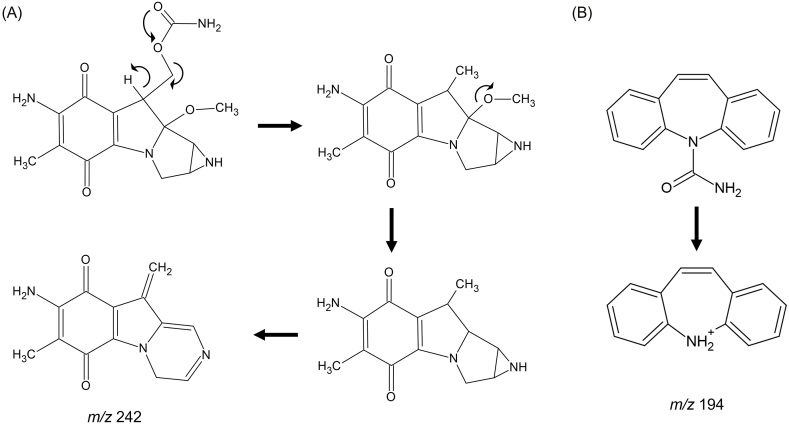
The precursor and product ion structure of MMC (A) and CBZ (B).

The chemical structures of the precursor and product ions of MMC and CBZ were depicted in Fig. 2 [36]. The product ion mass spectra of MMC and CBZ (IS) were displayed in Fig. 1. Under scan mode, MMC and IS yielded mostly ions [M + H]^+^ at *m*/*z* 335.2 and 237.1, respectively. The mass spectra's most prevalent fragmentation ion, in which the MMC product ion was discovered at *m*/*z* 242.3, was identified. Using the MRM for high selectivity and sensitivity of acquisition data in positive ESI mode, MMC and IS were quantified. Precursor to production transitions *m*/*z* 335.2 > 242.3 for MMC and *m*/*z* 237.1 > 194.4 for IS were utilized. The production of the fragment's transition from the particular precursor was optimized. A reproducible separation of MMC and IS was established after the chromatographic separation parameters had been properly adjusted.

### 3.2 Method validation

#### 3.2.1 Specificity and selectivity

**Fig. 3:**
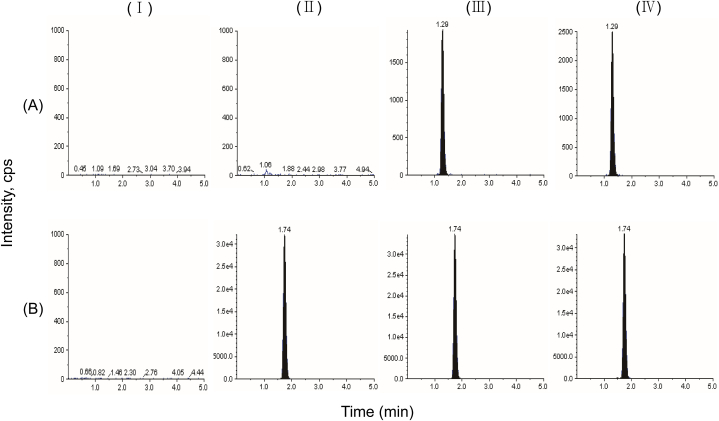
Representative chromatograms of MMC (A) and IS (B): (Ⅰ) blank plasma matrix, (Ⅱ) blank plasma matrix spiked with IS at 200 ng/mL, (Ⅲ) blank plasma spiked with LLOQ MMC conc and IS (Ⅳ) plasma sample after oral administration at 30 min.

To assess the specificity of the method, at least six normal individual blank plasma samples were analyzed to investigate the potential interference of analytes and IS in the liquid chromatography peak region. Our results showed that there were no significant interfering peaks observed in the retention times of MMC and are in blank matrix samples after following our method. The retention time for CBZ was 1.29 min, and for IS was 1.74 min. For one analysis, 5.0 min were required in total. Good analytes signal response, symmetric peak shapes, short run times, and good resolution can all be achieved with the suggested approach. This indicates that our method is highly selective and specific for detecting MMC. A representative chromatogram of the analytes in blank plasma, blank samples spiked with IS (200 ng/mL), blank plasma with MMC (10 ng/mL) and IS (200 ng/mL), and oral administration samples at 30 min were presented in Fig. 3, further supporting the specificity and selectivity of our method for detecting MMC.

#### 3.2.2 Carry-over

When injecting a blank sample right after the examination of a QCH sample, the carry-over impact was assessed. Our results showed that the peak responses at the retention time of analytes in the blank sample were less than 20 % of the LLOQ of MMC and 5 % of the IS response. This indicates that there was no significant carry-over effect observed in our method.

#### 3.2.3 Calibration curves and sensitivity

The calibration curves for MMC were constructed on five separate days and the back-calculated concentrations were found to be within 85–115 % for all concentration levels, except for 80–120 % for the LLOQ. The linear concentration range of MMC was determined to be 10–4000 ng/mL with a 1/x weighting factor. The coefficient of determination (r^2^) in standard calibration curves exhibited a mean value of 0.996 for MMC and ranged from 0.992 to 0.999. The LLOQ for MMC was determined to be 10 ng/mL, which provides adequate sensitivity for pharmacokinetic studies of MMC following oral and intravenous administration.

#### 3.2.4 Intra- and inter-run precision and accuracy and sample dilution linearity

The precision and accuracy of the MMC were evaluated in both intra-run and inter-run analyses over the course of three validation runs in order to validate the method. Each run included four concentration levels and five QC samples. Table 1 presents the results, which demonstrate that the MMC's accuracy and precision, both intra-day and inter-day, were within 15 % (and 20 % at LLOQ) of their nominal concentrations.

**Table 1:** Intra-day and inter-day precision and accuracy of plasma QC samples of MMC.

Nominal Concentration (ng/mL)	Measured Concentration (ng/mL)	Precision^a^ (RSD, %)	Accuracy^b^ (RE, %)
Intra-day batch (*n* = 5)			
10	10.4 ± 1.09	10.4	3.6
30	30.2 ± 2.85	8.4	4.4
1000	943 ± 90.3	7.1	6.3
4000	4056 ± 315	7.6	1.6
Inter-day batch (*n* = 5)			
10	10.5 ± 1.46	14.7	5.19
30	31.3 ± 4.20	14.3	4.42
1000	870 ± 56.9	7.01	13.0
4000	4130 ± 392	10.2	3.17

a Precision (%) = (standard deviation of concentration/mean concentration) × 100.

b Accuracy (%) = │100 – (mean concentration × 100/nominal concentration)│.

#### 3.2.5 Recovery and matrix effect

Matrix effects were investigated using the post-extraction spike method. The investigation of matrix effects revealed that both MMC and CBZ fell within the approved range according to FDA guidelines. (Table 2). For the recovery test, we compared the results obtained with and without matrix addition. The recovery of the analyte was consistent at both Low and High QC levels and the corresponding IS did not show a significant difference. Consequently, the IS proved effective in accurately tracking the analytes during the extraction process (Table 2). These outcomes indicate that any ion suppression or enhancement resulting from the rat plasma matrix was negligible in the present study.

**Table 2:** Matrix effect and recovery of MMC in rat plasma (n = 4).

Concentration (ng/mL)	Extraction Recovery		Matrix Effect	
	Mean (%)	RSD (%)	Mean (%)	RSD (%)
30	103	9.01	97.8	11.7
1000	104	7.92	1.02	7.39
4000	95.8	8.41	96.0	4.41
IS	98.1	12.5	1.11	8.06

#### 3.2.6 Stability

The stability of MMC in rat plasma was evaluated at low and high QC concentrations across diverse conditions. MMC remains stable in plasma for a minimum of 4 h at room temperature with an accuracy within ±4.8 %, and for a duration of 14 days at 4 °C, upholding an accuracy within ±9.0 %. Even after undergoing three cycles of freezing and thawing, no notable alteration (≤± 2.2 %) in MMC concentrations was not appreciable change. Long-term storage of plasma QC samples at −20 °C for 1 month did not result in any significant loss (≤± 12.3 %) of MMC. The stock solution stored for 1 day at room temperature and 7 days at 4 °C displayed unwavering stability with achieved accuracy values of ±3.4 %, ±13.5 % thus evincing no significant loss. A summary of these results is presented in Table 3.

**Table 3:** Stability of MMC in rat plasma (n = 4).

Experimental condition	Nominal Concentration (ng/mL)	Measured Concentration (ng/mL)	Precision (RSD, %)	Accuracy (RE, %)
4 h at room temperature	30	28.6 ± 2	5.4	4.8
	4000	3983 ± 234	5.9	0.4
14 days at 4 °C	30	27.3 ± 3	10.4	9.0
	4000	4135 ± 126	3.0	3.4
1 month at - 20 °C	30	33.7 ± 4	11.1	12.3
	4000	4210 ± 293	7.0	5.3
3 freeze-thaw cycles	30	30.7 ± 3	8.2	2.2
	4000	4043 ± 140	3.5	1.1
Post-preparation 5 days at 4 °C	30	28.3 ± 2	5.5	5.8
	4000	3698 ± 68	1.9	7.6
Stock solution stability, 1 day at RT	30	30.7 ± 5	14.7	2.3
	4000	3863 ± 82	2.1	3.4
Stock solution stability, 7 days at 4 °C	30	26.0 ± 1	2.1	13.5
	4000	4050 ± 260	6.4	1.3

### 3.3 Pharmacokinetic study

**Fig. 4:**
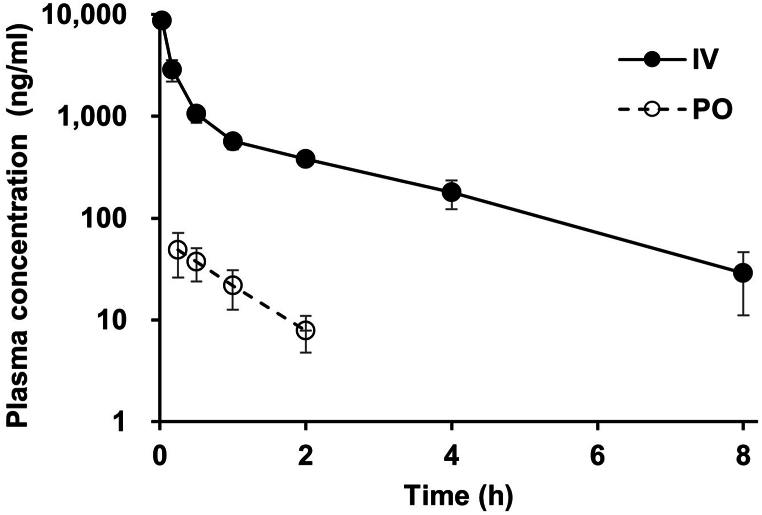
Mean plasma concentration-time curve following intravenous (black circle) and oral (white circle) administration at 5 mg/kg dose of MMC to SD rats. (mean ± standard deviation, n = 3 rats).

It proved affordable to successfully use the present verify method to the pharmacokinetic measurement of MMC in rats. The plasma concentration-time profiles of MMC after intravenous (5 mg/kg dose) and oral (5 mg/kg dose) administration to rats are shown in Fig. 4. Plasma was taken up to 8 h after the drug was administered intravenously and orally, and the drug's concentration was readily identifiable in samples. The calculated pharmacokinetic parameters such as C_max_, T_max_, t_1/2_, AUC_0→t_, AUC_0→∞_, V_ss_, CL, and F value are reported in Table 4. It was as able to detect MMC concentration in plasma samples up to 8 h after intravenous administration in rats. However, for oral administration, MMC concentrations in rat plasma were below the detection limit after 2 h. The results of these analyses showed low absorption and poor oral bioavailability of MMC. Although these pharmacokinetic results are from rat and cannot be directly applied them to humans, it can be estimated to humans after supplemented with extrapolation methods such as allometry scaling. This analytical approach can be applied to further studies of the toxicity and ADME/PK of MMC.

**Table 4:** The pharmacokinetic parameters of MMC following IV and oral administration at a dose of 5 mg/kg, to rats (n = 3). Data represent mean ± standard deviation.

Parameter	Unit	IV	PO
C_max_	ng/mL	–	50 ± 22
T_max_	hr	–	0.3 ± 1.4
*t*_1/2_	hr	1.4 ± 0.1	0.7 ± 0.0
AUC_0→t_	ng × hr/mL	3594 ± 350	54 ± 22
AUC_0→∞_	ng × hr/mL	3640 ± 354	62 ± 25
CL	L/kg/hr	1.4 ± 0.1	87.9 ± 50.7
V_ss_	L/kg	0.7 ± 0.1	–
F	%	–	1.5

C_max_, maximum peak concentration.

T_max_, time to reach C_max_.

t_1/2_, terminal half-life.

AUC_0→t_, area under the plasma concentration–time curve from time zero to the last time point.

AUC_0→∞_, area under the plasma concentration–time curve from time zero to infinity.

V_ss_, volume of distribution at steady state.

CL, elimination clearance.

F, the extent of absolute oral bioavailability.

## 4 Conclusions

With a calibration range of 10–4000 ng/mL, a selective and repeatable LC-MS/MS methodology has been developed and verified for the measurement of MMC in rat plasma. The result from the validation of the LC-MS/MS-based method shows good linearity over the calibration range, great accuracy and precision, high extraction recovery, and no matrix effect. The method fully matched with FDA guidelines for all validation parameters. Additionally, a pharmacokinetic study of MMC in rats following IV and oral administration was successfully conducted using this methodology. The oral bioavailability of MMC was 1.5 %. Based on the data reported in this study, this analytical method appears to be applicable for other rat studies of MMC to explain the chemotherapy of cancer using MMC like HIPEC.

## CRediT authorship contribution statement

**Kyung-Sun Moon:** Writing – review & editing, Writing – original draft, Visualization, Validation, Software, Methodology, Investigation, Formal analysis, Data curation, Conceptualization. **Jong-Min Song:** Methodology, Investigation, Formal analysis. **JiMin Yi:** Methodology, Investigation, Formal analysis. **Quynh Khoa Pham:** Writing – review & editing. **Sung-Hoon Ahn:** Writing – review & editing, Supervision, Resources, Project administration, Funding acquisition, Data curation, Conceptualization.

## Declaration of competing interest

The authors declare that they have no known competing financial interests or personal relationships that could have appeared to influence the work reported in this paper.
